# Recurrent Stroke after Transcatheter PFO Closure in Cryptogenic Stroke or Tia: Long-Term Follow-Up

**DOI:** 10.1155/2017/9849425

**Published:** 2017-12-21

**Authors:** Elisabetta Mariucci, Andrea Donti, Luisa Salomone, Marta Marcia, Marta Guidarini, Roberto Formigari, Daniela Prandstraller, Anna Balducci, Gabriele Bronzetti, Marco Bonvicini

**Affiliations:** Pediatric Cardiology and GUCH Unit, S. Orsola-Malpighi Hospital, University of Bologna, 9 Massarenti Street, 40100 Bologna, Italy

## Abstract

**Background:**

There are few data on the mechanism of recurrent neurological events after transcatheter closure of patent foramen ovale (PFO) in cryptogenic stroke or TIA.

**Methods:**

We retrospectively reviewed PFO closure procedures for the secondary prevention of cryptogenic stroke/TIA performed between 1999 and 2014 in Bologna, Italy.

**Results:**

Written questionnaires were completed by 402 patients. Mean follow-up was 7 ± 3 years. Stroke recurred in 3.2% (0.5/100 patients-year) and TIA in 2.7% (0.4/100 patients-year). Ninety-two percent of recurrent strokes were not cryptogenic. Recurrent stroke was noncardioembolic in 69% of patients, AF related in 15% of patients, device related in 1 patient, and cryptogenic in 1 patient. AF was diagnosed after the procedure in 21 patients (5.2%). Multivariate Cox's proportion hazard model identified age ≥ 55 years at the time of closure (OR 3.16, *p*=0.007) and RoPE score < 7 (OR 3.21, *p*=0.03) as predictors of recurrent neurological events.

**Conclusion:**

Recurrent neurological events after PFO closure are rare, usually noncryptogenic and associated with conventional vascular risk factors or AF related. Patients older than 55 years of age and those with a RoPE score < 7 are likely to get less benefit from PFO closure. After transcatheter PFO closure, lifelong strict vascular risk factor control is warranted.

## 1. Introduction

Stroke is the second most common cause of death and the leading cause of disability worldwide. Approximately 87% of all strokes are ischemic [1]. According to the TOAST classification [2], about 30% of strokes are due to large artery atherosclerosis, 20% are cardioembolic, 15% are due to small artery occlusion (lacunar), 5% have other determined etiology (such as nonatherosclerotic vasculopathies, hypercoagulable states, or hematologic disorders), and about 30% of strokes have no identifiable cause even after extensive evaluation and are designated cryptogenic stroke (CS).

Establishing the etiology of a stroke has profound implications for subsequent treatment and the risk of recurrent events.

The reported rate of recurrent stroke in patients with CS varies widely, averaging 3–6% per year [3]. Young patients with CS associated to a patent foramen ovale (PFO) have stroke recurrence rates of 1–2% per year when given aspirin [4, 5]. The recurrence rate is substantially higher in older patients (14% per year in one report), suggesting the roles of other causes in addition to paradoxical embolism [6].

The rate of recurrent stroke after transcatheter closure of PFO in the RESPECT trial was 0.66% per year [7]. Stroke or TIA recurrence was 1.06% per year in the PC trial [8] and 2.6% per year in the CLOSURE I trial [9]. Recently, Taggart et al. [10] reported an annualized recurrence rate of stroke of 0.5% per year and an annualized recurrence rate of either stroke or TIA of 1.0% per year.

There are few data on the mechanism of recurrent neurological events after transcatheter closure of PFO in CS or TIA. RESPECT investigators in the extended follow-up analysis observed that nearly 1/3 of recurrent strokes in the Amplatzer PFO Occluder arm were not cryptogenic but of known origin.

The aim of our study was to establish the mechanism of recurrent neurological events during long-term follow-up after transcatheter closure of PFO in CS or TIA.

## 2. Methods

All PFO closure procedures performed between April 1999 and March 2014 for the secondary prevention of CS/TIA at the Pediatric Cardiology and GUCH Unit of S. Orsola-Malpighi Hospital in Bologna, Italy, were retrospectively reviewed. Demographic, clinical, procedural, and follow-up data were evaluated. Questionnaires and written consent forms were sent to all patients. All patients who gave written informed consent were subsequently contacted by telephone and included in the study. The study was approved by the S. Orsola-Malpighi Hospital Institutional Review Board. Patients who underwent PFO closure for reasons other than the secondary prevention of stroke/TIA and those who did not provide written informed consent were not included in this study.

All patients with recurrent stroke/TIA or a diagnosis of atrial fibrillation (AF) were invited to return to the clinic for an examination. Clinical history, hospitalization reports, neurological examination reports, and imaging studies were obtained. The etiology of recurrent neurological events was investigated, and data were analyzed to identify risk factors. We retrospectively applied the RoPE score [11] to our study population to evaluate the relationship of recurrent events to the likelihood that the index event was PFO related. We also retrospectively applied to our population other scores used for patients with AF (CHA2DS2-VASc score [12], HATCH score [13], and ATRIA score [14]) to evaluate any relationship with recurrent neurological events.

### 2.1. Patient Selection for Device Closure

Patients were eligible for device closure if they had a cryptogenic ischemic stroke or TIA and a PFO identified by transesophageal echocardiography (TEE). Ischemic stroke and TIA were defined according to the AHA/ACC guidelines [15].

Patent foramen ovale was defined on the basis of transesophageal echocardiographic evidence of a separation between the septum primum and septum secundum, with the demonstration of right-to-left shunt by contrast injection [16]. Detecting right-to-left shunt by contrast was defined as echocardiographic evidence of infused microbubbles in the left atrium within three cardiac cycles after their appearance in the right atrium, at rest or during Valsalva release. The shunt size was graded as conventionally accepted [16]. Atrial septal aneurysm (ASA) was defined as a protrusion of septal tissue of >10 mm from the plane of the atrial septum into either the right or the left atrium evaluated by means of TEE.

From 2003 onwards, all patients (307) underwent transcranial Doppler (TCD) ultrasonography to quantify right-to-left shunt before and after device closure. Shunt size was graded on a modified scale based on the “Venice Consensus Conference” and more recently available literature reports [17–20]: 0 to 10 microbubbles indicating no shunt, 10 to 25 microbubbles mild shunt, 26 to 50 microbubbles moderate shunt, and more than 50 microbubbles or a shower or curtain of microbubbles severe shunt. Before 2003, right-to-left shunt was evaluated by means of contrast TEE before device closure and contrast transthoracic echocardiography (TTE) after device closure and maximum shunt size was reported (95 patients).

All patients with ischemic stroke or TIA underwent an extensive neurologic and cardiological evaluation in order to diagnose CS or TIA. The examinations carried out were brain CT and MRI, ultrasonography of cervical arteries plus TCD ultrasonography of intracranial vessels or magnetic resonance angiography (MRA) or computerized tomographic angiography (CTA) of the head and neck, 24-hour Holter monitoring to exclude atrial fibrillation, TTE and TEE to exclude potential causes of cardioembolism, and screening for thrombophilic states. Patients complaining of palpitations underwent prolonged intermittent ECG monitoring with an external event recorder and were invited to record their ECG during symptoms. The decision to proceed with device closure was made by the cardiologist after consultation with the neurologist. Patients were excluded from the analysis if a mechanism of the index ischemic event other than paradoxical embolization could be identified.

### 2.2. Procedure

The implantation procedures were performed under general anesthesia and transesophageal guidance. All patients received oral aspirin (100 mg/day) plus clopidogrel (75 mg/day) or ticlopidine (250 mg twice a day), starting at least three days prior to the procedure. During the procedure, all patients underwent anticoagulation with intravenous heparin (100 U/kg) and received antibiotic prophylaxis with cefuroxime (1.5–2 g IV). Device type and size were selected according to PFO anatomy at the discretion of the cardiologist performing the procedure. Contrast TEE was performed at the end of the procedure to assess residual shunt.

The devices used were Amplatzer PFO occluder, Amplatzer septal occluder, and Amplatzer cribriform devices (St. Jude Medical), Gore HELEX septal occluder (W.L. Gore & Associates), Premere PFO occluder (St. Jude Medical), CardioSEAL septal occluder and BioSTAR septal occluder (NMT Medical), Cardia Star and Atriasept septal occluder (Cardia), Occlutech Figulla flex II (Occlutech), and Nit-Occlud PFO occluder (pfm medical). The CardioSEAL and BioSTAR device were no longer implanted after 2005.

Postprocedure TTE was performed prior to hospital discharge, usually the day after the procedure, to confirm device position and exclude pericardial effusion. All patients received oral aspirin (100 mg/day) plus clopidogrel (75 mg/day) or ticlopidine (250 mg twice a day) for the first 3 months, followed by aspirin alone for 9 months. Subsequently, antiplatelet therapy was administered to all patients with a residual shunt. Most of the patients with no residual shunt but with vascular risk factors continued aspirin. Patients who needed oral anticoagulation for other reasons continued this therapy. In accordance with ESC guidelines [21, 22], prophylaxis for bacterial endocarditis was recommended after defect closure.

### 2.3. Follow-Up

Cardiological follow-up examinations were performed 3 and 12 months after procedure and yearly thereafter. All patients underwent TCD ultrasonography or TTE with saline contrast injection 3 and 12 months after procedure to quantify residual right-to-left shunt. If a significant residual shunt was detected at 1 year, TEE was performed to check whether a second device was needed. Patients who experienced a recurrent neurological event underwent TEE to evaluate device position, residual shunt, and intracardiac thrombus. Two years after PFO closure, patients not residing in Bologna continued cardiology follow-up at home.

Follow-up events were recurrent neurological or peripheral thromboembolic events, the need for reintervention in the case of a significant residual shunt or device misalignment, device erosion or embolization, and the onset of tachyarrhythmias. Tachyarrhythmias after PFO closure were detected by 12-lead ECG (performed at each follow-up visit), 24-hour ECG Holter monitoring, and external intermittent event recorder in patients complaining of palpitations or with recurrent stroke/TIA.

### 2.4. Statistical Analysis

Continuous descriptors were expressed as mean and standard deviation (SD) and compared by using the two-sided unpaired Student's *t*-test. The categorical descriptors were summarized as frequencies and percentages and compared by using the two-tailed chi-square test.

Recurrent stroke and TIA and rates of AF detection during follow-up were shown by means of Kaplan–Meier plots and were compared by means of a log-rank test.

The association of recurrent stroke and TIA or AF detection during follow-up with baseline characteristics was tested by computing their odds ratio (OR) and compared by two-tailed Fisher's exact test. The multivariate Cox's proportion hazard model (with forward stepwise option) was used to select independent predictors of events and to calculate their Hazard Ratio.

Statistical significance was based on a two-sided type I error rate of 0.05. All statistical tests were performed by means of IBM-SPSS, version 21.0 (IBM-SPSS, Chicago, Illinois, USA, 2012).

## 3. Results

### 3.1. Baseline Characteristics

Between April 1999 and March 2014, 525 patients underwent device PFO closure for the secondary prevention of CS/TIA at the Pediatric Cardiology and GUCH Unit of S. Orsola-Malpighi Hospital in Bologna, Italy. Long-term follow-up data were obtained by means of written questionnaires in 402 patients.

All but one patient with recurrent stroke/TIA or AF diagnosis returned to the clinic for examination.

The primary indication for PFO closure was stroke in 78%, TIA in 15%, systemic peripheral embolism in 1%, and silent brain infarction in 6%. Neuroimaging revealed multiple infarcts in 28% of patients and bilateral lesions in 25%. Mean age at the time of the index event was 47 ± 13 years. Sixty-one patients had had a previous stroke or TIA (15%) and 1 patient had a previous systemic peripheral embolism episode. Mean RoPE score at the baseline evaluation in the PFO clinic was 6 ± 2; 38% of patients presented a RoPE score ≥ 7. Baseline clinical characteristics of this cohort are shown in Table 1.

Baseline TEE evaluation documented an ASA in 210 patients (52%). Preclosure right-to-left shunt was severe in 95% of patients, as summarized in Table 2.

### 3.2. Procedure

Mean age at the time of the procedure was 48 ± 13 years. The most frequently used device was the Amplatzer PFO occluder (209 patients, 52%). The mean diameter of the device implanted was 25 ± 5 mm (range 10–35 mm). Contrast TEE performed at the end of the procedure revealed residual right-to-left shunt in 26% of patients: it was mild in 85% of cases and moderate in 15%.

Procedure- and device-related complications occurred in 31 patients (7.7%): major complications occurred in 0.2% of patients and minor complications in 7.5%.

There were no strokes or deaths associated with device placement. No device embolization was observed. Four patients had a device implanted and released which was then felt to be inadequately seated: in 3 cases the device was successfully removed and replaced with a larger device, whereas 1 patient needed surgical explantation of the device. Two patients (0.5%) developed AF or flutter during hospitalization.

On discharge, 78% of patients received dual antiplatelet therapy, 17% received oral anticoagulants, and 5% received a single antiplatelet agent. Single antiplatelet therapy was administered only to patients with aspirin allergy, groin hematoma, postprocedural epistaxis, or hemoptysis and the patient who was submitted to surgical operation.

### 3.3. Echocardiographic Follow-Up

The last clinical and echocardiographic follow-up examination in Bologna was performed 2 ± 0.5 years after procedure. No patient experienced device embolization or malpositioning or device-related valve dysfunction.

Significant (moderate or severe) residual right-to-left shunt was observed in 6% of patients. Residual right-to-left shunt grade at 12-month follow-up examination is summarized in Table 2.

All patients with evidence of a severe residual shunt at 12 months were scheduled for reintervention. Twelve patients underwent implantation of a second device, and 1 patient was diagnosed with an arteriovenous pulmonary fistula which was embolized.

Of the 12 patients who underwent second device implantation, 7 presented complete shunt closure, 3 mild residual shunt, and 2 moderate residual shunt on 12-month follow-up examination.

One patient presented a mild pericardial effusion which spontaneously resolved in few weeks.

### 3.4. Short-Term Follow-Up

One patient suffered a recurrent stroke 2 weeks after PFO closure: TCD ultrasonography showed no residual right-to-left shunt, TEE revealed no left atrium or left disk thrombosis, and no arrhythmias were documented by prolonged ECG monitoring. He had stopped dual antiplatelet therapy the day after PFO closure because of copious epistaxis related to nasogastric tube positioning during the procedure, and restarted aspirin only 2 days later. Device thrombosis was suspected, and the patient was treated with oral anticoagulation for 6 months; subsequently, on single antiplatelet therapy, he did not suffer further recurrent neurological events.

One patient who had undergone Cardio-SEAL device implantation was studied with a TEE 1 month after the procedure as part of an internal protocol and was found to have a thrombus on the left disk. He was treated with oral anticoagulation for 12 months and subsequently with single antiplatelet therapy for residual shunt. He did not present neurological events on follow-up.

One patient on oral anticoagulation developed subdural hematoma 4 months after the procedure.

### 3.5. Long-Term Clinical Follow-Up

**Figure 1:**
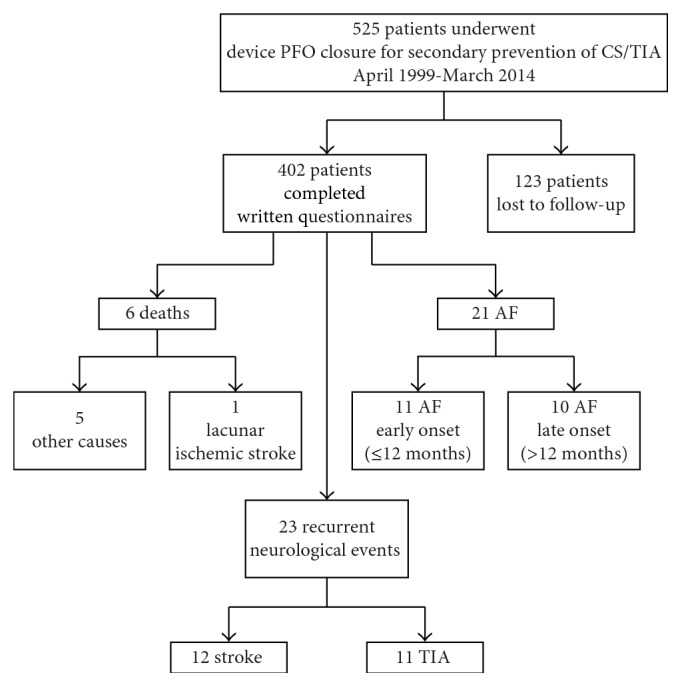
Flowchart demonstrating events at follow-up.

Mean long-term follow-up was 7 ± 3 years (range 2–16).  Figure 1 is a flowchart demonstrating events at follow-up.

There were 6 deaths, none due to a device-related complication. Four deaths were attributed to cancer, one to infection, and one to lacunar ischemic stroke. No device-related complications (embolization, erosion, and pericarditis) were recorded.

Recurrent neurological events occurred in 24 patients (6%): stroke in 13(3.2%) and TIA in 11 (2.7%). Recurrent stroke/TIA occurred 4 ± 3.7 years after PFO closure (range 2 weeks–13 years).

**Figure 2:**
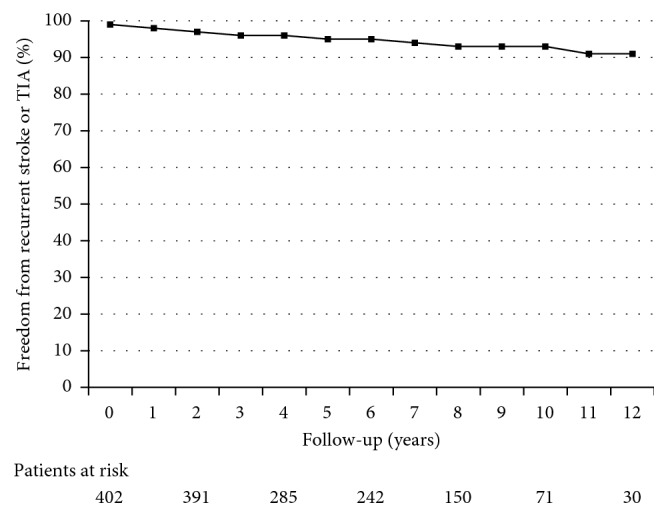
Kaplan–Meier plot showing freedom from stroke or TIA over a 10-year time frame.

The recurrence rate of stroke was 0.5/100 patients-year, that of TIA was 0.4/100 patients-year, and that of either stroke or TIA was 0.86/100 patients-year. Freedom from recurrent stroke was 99.5% at 1 year, 97.9% at 5 years, and 96.1% at 10 years. Kaplan–Meier plot showing freedom from recurrent stroke or TIA is reported in Figure 2.

Patients with recurrent neurological events were older at the time of PFO closure (54 ± 12 versus 48 ± 13 years old, *p*=0.013) and more frequently had hypertension (69% versus 39%; *p*=0.042). Sixty-nine percent of patients with recurrent stroke presented a basal RoPE score < 7 before PFO closure.

Recurrent neurological events were more frequent in subjects with RoPE score (risk of paradoxical embolism) < 7 than in those with ≥7 (8% versus 2.6%; OR 3.21, *p*=0.03) as summarized in Table 3.

Multivariate Cox's proportion hazard model identified age ≥ 55 years at the time of closure (OR 3.16, *p*=0.007) and RoPE score < 7 (OR 3.21, *p*=0.03) as predictors of recurrent neurological events (Table 4). PFO closure performed with the Amplatzer device resulted a protective factor (OR 0.39, *p*=0.03). No association was observed between residual shunt and recurrent events.

**Figure 3:**
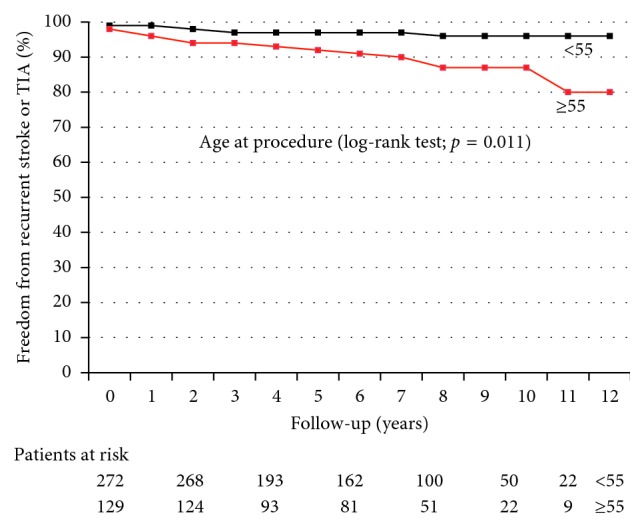
Kaplan–Meier plot showing freedom from recurrent stroke and/or TIA over a 10-year time frame in patients aged ≥ 55 years versus < 55 years at the time of the procedure. *p*=0.011 by log-rank test.

**Figure 4:**
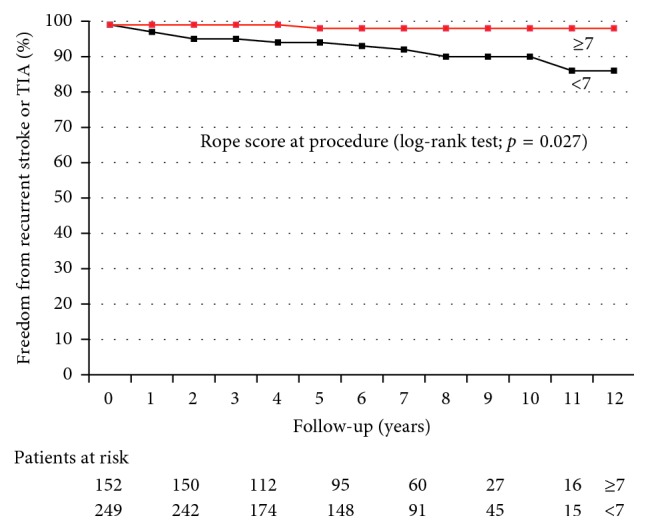
Kaplan–Meier plot showing freedom from recurrent stroke and/or TIA over a 10-year time frame in patients with RoPE score < 7 versus those with RoPE score ≥ 7 at the time of the procedure. *p*=0.027 by log-rank test.

The Kaplan–Meier plot showing freedom from recurrent stroke and/or TIA over a 10-year time frame in patients aged ≥ 55 years versus <55 years at the time of the procedure is reported in Figure 3. The Kaplan–Meier plot showing freedom from recurrent stroke and/or TIA over a 10-year time frame in patients with RoPE score < 7 versus those with RoPE score ≥ 7 at the time of the procedure is reported in Figure 4.

All patients with recurrent stroke or TIA returned to the clinic for examination, except one, for whom only the brain imaging report was available, and the etiology of recurrent stroke could not be investigated (patient number 3). Patient characteristics of recurrent stroke group are shown in Table 5. Recurrent stroke was noncardioembolic (lacunar or atheroembolic) in 69% of patients, AF related in 15%, device related in 1 patient, and possibly cryptogenic in 1 patient (patient number 3, who had no residual shunt, inadequate control of multiple vascular risk factors, and in whom AF was not investigated). Device-related stroke occurred in the patient who stopped dual antiplatelet therapy the day after the procedure because of copious epistaxis (patient number 9).

### 3.6. AF after PFO Closure

During the follow-up period, 21 patients (5.2%) developed AF. AF was persistent in 2 patients and paroxysmal in the others and was discovered after a median time of 3 months (range 0 days–8 years). The mean age on arrhythmia diagnosis was 61 ± 10 years (range 44–73 years). Patients who developed AF were older at the time of PFO closure (60 ± 10 versus 47 ± 13 years, *p*<0.001) and more frequently had hypertension (69% versus 39%; *p*=0.016). Multivariate Cox's proportional hazards analysis showed age ≥ 55 years at the time of closure (OR 7.49, *p*=0.001) as the main risk factor for AF diagnosis on follow-up, as summarized in Table 6. Also, atherosclerotic cardiovascular disease (OR 2.99, *p*=0.04) and HATCH score ≥ 3 at the time of closure (OR 3.09, *p*=0.02) resulted risk factors for AF diagnosis on follow-up.

Eleven patients (2.7%) developed AF within 12 months after PFO closure: 6 received antiarrhythmic therapy for 12 months and suffered no further recurrence, 4 were treated with chronic antiarrhythmic therapy, and 1 patient with permanent AF underwent chronic pharmacological rate control.

The remaining 10 patients (2.5%) developed AF more than 12 months after PFO closure (range 18 months–8 years).

**Figure 5:**
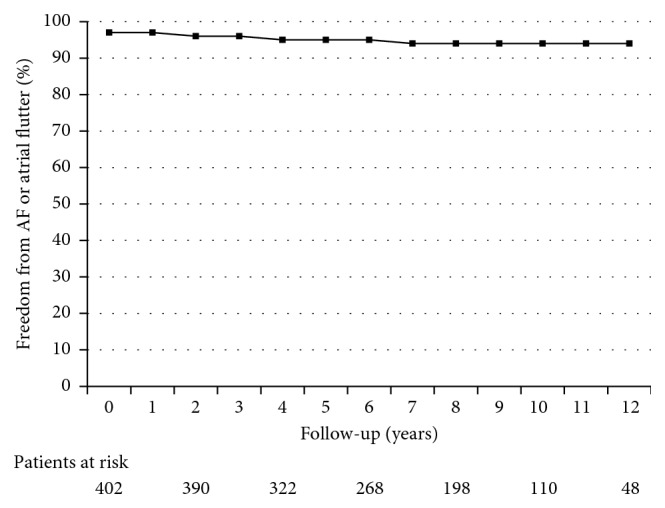
Kaplan–Meier plot showing freedom from AF over a 10-year time frame.

**Figure 6:**
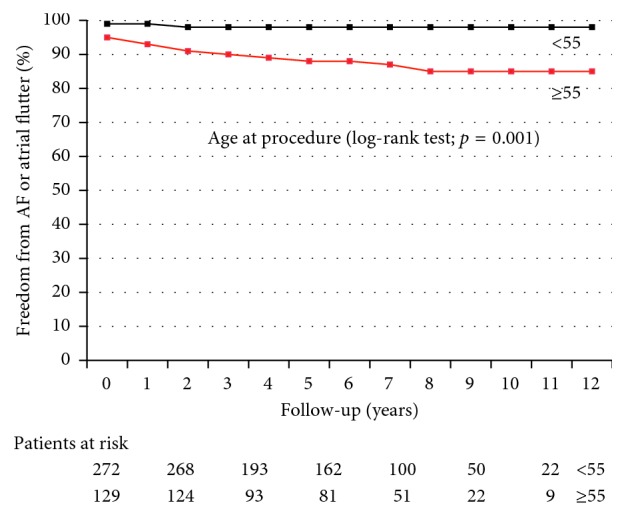
Kaplan–Meier plot showing freedom from AF over a 10-year time frame in patients aged ≥ 55 years versus < 55 years at the time of the procedure. *p*=0.001 by log-rank test.

The Kaplan–Meier plot showing freedom from AF is reported in Figure 5. Freedom from AF was 97.3% at 1 year, 95.3% at 5 years, and 94.7% at 10 years. The Kaplan–Meier plot showing freedom from AF over a 10-year time frame in patients aged ≥ 55 years versus < 55 years at the time of the procedure is shown in Figure 6.

## 4. Discussion

We investigated the etiology of recurrent neurological events in our single-center cohort of patients who had undergone transcatheter PFO closure for the secondary prevention of CS/TIA. The recurrence rate of stroke or TIA that we observed is relatively low being less than 1% per year. Moreover, as recently shown in other reports and in the extended follow-up analysis of the Respect trial, recurrent neurological ischemic events were usually noncryptogenic, mainly lacunar, and were associated with conventional vascular risk factors or AF related.

A good rate of functional PFO closure was observed, with an incidence of significant (moderate or severe) residual right-to-left shunt of 6%.

Our analysis shows a higher risk of recurrent neurological events after PFO closure in patients aged 55 years or older at the time of the procedure and those with a basal RoPE score < 7. This finding strongly suggests that in older patients with risk factors for cerebrovascular disease the probability that neurological events could be due to paradoxical embolism across a PFO is low. Therefore, these patients should not undergo PFO closure as they are very likely to get less benefit from the procedure.

Like other investigators [11, 23], we observed that the individuals most likely to benefit from PFO closure were younger (<55 years old), with a RoPE score ≥ 7, meaning they have no other identifiable stroke risk factor, and with a neurological imaging strongly in favour of a cardioembolic event. As 95% of all our patients had a severe right-to-left shunt prior to PFO closure, we cannot demonstrate any possible impact of shunt size on recurrent neurological events.

Of note, AF was rather common in the follow-up of our patients after PFO closure (5.2%). Only a minority of the cases are probably directly due to the device as many patients developed AF more than 12 months after the procedure or needed prolonged antiarrhythmic therapy to avoid recurrences. Moreover, AF was more common in older and hypertensive patients, suggesting that patient's features are more relevant than device implantation in AF occurrence. As we did not routinely screen for AF with prolonged ECG monitoring before PFO closure, we cannot rule out AF as the possible cause of the index event too. Nevertheless, we recognize the crucial potential role of prolonged ECG monitoring before PFO closure, and we are currently screening all patients in such a way.

## 5. Limitations

Our study was retrospective, and our long-term clinical follow-up data after PFO closure were obtained by means of written questionnaires. Although we used telephone interviews to verify the accuracy of the data obtained, this approach introduces the potential for a recall bias.

Moreover, the patients' baseline RoPE scores were calculated retrospectively, which may have caused an underestimation of the true scores.

Patient selection was carried out before the publication of the RoPE score, and we realize that some patients who underwent PFO closure may have had an incidental PFO and not a PFO-mediated stroke.

In addition, as patient selection for PFO closure was undertaken before the publication of strong evidence of the utility of prolonged ECG monitoring to detect silent AF after CS [15, 24–27], a thorough search for atrial arrhythmias was not performed. Some patients may therefore have been misclassified as having had a CS, while a more complete evaluation may have revealed the underlying silent AF.

We identified PFO closure performed with Amplatzer device as a protective factor against recurrent neurological events. We could not demonstrate any possible impact of other devices on recurrent neurological events because of the low number of devices implanted.

Finally, the main limitation of our study, as in the case of many similar studies, is the small number of recurrent neurological events, in which yielded a low statistical power to identify potential risk factors for recurrences.

## 6. Conclusion

Recurrent neurological events after PFO closure are rare, usually noncryptogenic and associated to conventional vascular risk factors or AF related. Patients older than 55 years of age and those with a RoPE score < 7 are likely to get less benefit from PFO closure. After transcatheter PFO closure, lifelong strict vascular risk factor control is warranted.

## Figures and Tables

**Table 1:** Baseline clinical characteristics of patients before PFO closure (*n* = 402).

	*N*	%
Male	209	52.0
Family history of ASCVD	91	22.6
Cigarette smoking	88	21.4
Former smoker	85	21.1
Dyslipidemia	141	35.1
Hypertension	117	29.1
Diabetes mellitus	8	2.0
Overweight or obesity	47	11.7
ASCVD	51	12.7
Oral contraceptives	36	9.0
Thrombophilia	114	28.6
Prior PE and/or DVT	19	4.7
Prior stroke/TIA (before index event)	61	15.0
Migraine headache	130	32.3
Migraine headache with aura	88	21.9
Palpitations	10	3.6
Index event of stroke	314	78
Cortical infarct on imaging	185	46
RoPE score ≥ 7	152	38
CHA2DS2-Vasc score ≥ 3	251	62
HATCH score ≥ 3	111	28
ATRIA score ≥ 9	244	61

AF, atrial fibrillation; ASCVD, atherosclerotic cardiovascular disease; ATRIA score, Anticoagulation and Risk Factors in AF score [14]; CHA2DS2-VASc score, Congestive heart failure, Hypertension, Age ≥75 years (doubled), Diabetes mellitus, Prior Stroke or TIA or thromboembolism (doubled), Vascular disease, Age 65–74 years, Sex category score [12]; DVT, deep vein thrombosis; HATCH score, Risk Factors for Progression to Persistent AF score [13]; *n*, number; PE, pulmonary embolism; PFO, patent foramen ovale; RoPE score, Risk of Paradoxical Embolism score [11].

**Table 2:** Preclosure and residual right-to-left shunt grade at 12-month follow-up (*n* = 402).

Baseline right-to-left shunt grade	Preclosure, *N* (%)	Residual, *N* (%)
Absent	114 (28)	360 (90)
Mild	99 (25)	33 (8)
Moderate	59 (15)	7 (1.6)
Severe	130 (32)	2 (0.4)

Valsalva right-to-left shunt grade	*N* (%)	*N* (%)

Absent	0	281 (70)
Mild	0	96 (24)
Moderate	20 (5)	11 (2.6)
Severe	382 (95)	14 (3.4)

Baseline evaluation revealed severe right-to-left shunt before device closure in 95% of patients. Significant (moderate or severe) residual right-to-left shunt was observed in 6% of patients. *N*, number; TCD, transcranial Doppler; TEE, transesophageal echocardiography; TTE, transthoracic echocardiography.

**Table 3:** Subgroup analysis of incidence of recurrent ischemic neurological events.

Risk score category	Recurrent stroke/TIA (*n*)	Recurrent stroke/TIA (%)	HR (95% CI)	*p* value
RoPE 7–10	4	2.6	3.21	0.03
RoPE 0–6	20	8		
CHA2DS2-Vasc 0–2	6	4	1.87	0.27
CHA2DS2-Vasc 3–7	18	7.2		
HATCH 0–2	11	4.5	2.35	0.06
HATCH 3–4	13	9.9		
ATRIA 0–8	6	3.8	2.02	0.19
ATRIA 9–15	18	7.4		

Recurrent neurological events were more frequent in subjects with RoPE (Risk of Paradoxical Embolism) score < 7 than those with ≥ 7 (OR 3.21, *p*=0.03).

**Table 4:** Predictors of recurrent ischemic neurological events.

	No. ischemic recurrence *n* (%)	Ischemic recurrence *n* (%)	OR	*p* value
Age at PFO closure ≥ 55 years	116 (30.7)	14 (58.3)	3.16	0.007
Male	197 (52.1)	12 (50.0)	0.91	1.00
Cigarette smoking	156 (41.3)	15 (62.5)	2.37	0.054
Dyslipidemia	130 (34.4)	11 (45.8)	1.60	0.27
Hypertension	106 (28.0)	11 (45.8)	2.17	0.10
Diabetes mellitus	8 (2.1)	0	—	1.00
BMI > 25 Kg/m^2^	43 (11.4)	4 (16.7)	1.56	0.51
ASCVD	46 (12.2)	5 (20.8)	1.53	0.21
Index stroke	348 (92.1)	24 (100)	—	1.0
AF diagnosis at follow-up	19 (5.0)	2 (8.3)	1.72	0.36
Atrial septal aneurysm	195 (51.6)	15 (62.5)	1.56	0.40
Severe R→L shunt before device closure	373 (98.7)	24 (100)	1.10	1.00
Residual significant R→L shunt^∗^	14 (3.7)	0	0.92	1.00
RoPE score < 7	230 (60.8)	20 (83.3)	3.21	0.03
CHA2DS2-Vasc score ≥ 3	233 (61.6)	18 (75.0)	1.87	0.28
HATCH score ≥ 3	100 (26.5)	11 (45.8)	2.35	0.06
ATRIA score ≥ 9	226 (59.8)	18 (75.0)	2.02	0.19
Amplatzer devices	228 (60.3)	9 (37.5)	0.39	0.03

Multivariate Cox's proportion hazard model identified age ≥ 55 years at the time of closure and RoPE score < 7 as predictors of recurrent neurological events. PFO closure performed with Amplatzer device resulted a protective factor. No association was observed between residual shunt and recurrent events. ^∗^Residual significant (moderate or severe) R→L shunt: the last TCD ultrasonography or TTE with saline contrast injection was used to investigate the association between residual shunt and recurrent events. AF, atrial fibrillation; ASCVD, atherosclerotic cardiovascular disease; BMI, body mass index; L, left; R, right; TIA, transient ischemic attack.

**Table 5:** Patient characteristics of recurrent stroke group (*n* = 13).

	Age on recurrent event (years)	Timing of recurrent stroke (years)	Index event brain lesion topography	Recurrent brain lesion topography	Therapy on recurrent stroke	Basal RoPE score	RoPE score on recurrent stroke	No. of vascular risk factors	Residual shunt	Residual shunt grade	Stroke etiology
1	66	8.2	Single subcortical	Lacunar	Single antiplatelet	4	2	4	No	—	Not cardioembolic
2	47	13.3	None	Lacunar	None	7	6	0	No	—	Not cardioembolic
3	60	0.6	Multiple subcortical	Cortical	None	2	3	3	No	—	CS
4	49	2.2	Multiple cortico-subcortical	Lacunar	Single antiplatelet	7	5	3	Yes	Moderate	Not cardioembolic
5	46	5.1	Single cortico-subcortical	Multiple cortico-subcortical	Single antiplatelet	7	4	2	No	—	Atheroembolic (homolateral carotid stenosis)
6	54	3.0	Multiple subcortical	Lacunar	None	5	3	4	No	—	Not cardioembolic
7	72	6.2	Multiple cortico-subcortical	Lacunar	Single antiplatelet	5	3	2	Yes	Mild	Not cardioembolic
8	61	5.2	Single subcortical	Lacunar	Single antiplatelet	5	3	3	Yes	Mild	Not cardioembolic
9	22	0	Multiple cortico-subcortical	Multiple cortico-subcortical	Single antiplatelet	10	9	0	No	—	Cardioembolic (presumed LA thrombosis)
10	62	2.3	Multiple cortico-subcortical	Multiple cortico-subcortical	Single antiplatelet	4	4	3	No	—	Cardioembolic (AF)
11	75	1.5	Multiple cortico-subcortical	Lacunar	None	4	3	2	No	—	Not cardioembolic
12	60	1.6	Single cortico-subcortical	Multiple cortico-subcortical	Single antiplatelet	6	4	2	No	—	Atheroembolic (homolateral carotid stenosis)
13	71	2.0	Multiple cortico-subcortical	Multiple cortico-subcortical	Single antiplatelet	3	3	3	Yes	Mild	Cardioembolic (AF)

All patients with recurrent stroke returned to the clinic for examination, except one patient, for whom only brain imaging was available, and the etiology of recurrent stroke could not be investigated (patient number 3). Recurrent stroke was noncardioembolic (lacunar or atheroembolic) in 69% of patients, AF related in 15% of patients, device related in 1 patient, and possibly cryptogenic in 1 patient (patient number 3, who had no residual shunt, inadequate control of multiple vascular risk factors, and in whom AF was not investigated). Device-related stroke occurred in patient number 9 who stopped dual antiplatelet therapy the day after the procedure because of copious epistaxis. AF, atrial fibrillation; CS, cryptogenic stroke, LA, left atrium, *N*, number.

**Table 6:** Predictors of AF or atrial flutter diagnosis on follow-up.

	No AF/Fla, *n* (%)	AF/Fla, *n* (%)	OR	*p* value
Age at PFO closure ≥ 55 years	114 (29.9)	16 (76.2)	7.49	0.001
Male	199 (52.2)	10 (47.6)	0.83	0.82
Cigarette smoking	163 (42.8)	8 (38.1)	0.83	0.83
Dyslipidemia	131 (34.4)	10 (47.6)	1.73	0.27
Hypertension	107 (28.1)	10 (47.6)	2.33	0.08
Diabetes mellitus	7 (1.8)	1 (4.8)	2.67	0.35
BMI > 25 Kg/m^2^	45 (11.8)	2 (9.5)	0.79	1.0
ASCVD	45 (11.8)	6 (28.6)	2.99	0.04
Index stroke	351 (92.1)	21 (100)	—	1.0
Atrial septal aneurysm	196 (51.4)	14 (66.7)	1.89	0.19
Severe R→L shunt before device closure	376 (98.7)	21 (100)	1.06	1.00
Residual significant R→L shunt	14 (3.7)	0	0.95	1.00
RoPE score < 7	233 (61.2)	17 (81.0)	2.70	0.10
CHA2DS2-Vasc score ≥ 3	234 (61.4)	17 (81.0)	2.67	0.10
HATCH score ≥ 3	100 (26.2)	11 (52.4)	3.09	0.02
ATRIA score ≥ 9	227 (59.6)	17 (81.0)	2.88	0.06
Amplatzer devices	226 (59.3)	11 (52.4)	0.75	0.65

Multivariate Cox's proportional hazards analysis showed age ≥ 55 years at the time of closure (OR 7.49, *p*=0.001) as the main risk factor for AF diagnosis on follow-up. Also, atherosclerotic cardiovascular disease (OR 2.99, *p*=0.04) and HATCH score ≥ 3 at the time of closure (OR 3.09, *p*=0.02) resulted risk factors for AF diagnosis on follow-up. AF, atrial fibrillation; ASCVD, atherosclerotic cardiovascular disease; BMI, body mass index; Fla, atrial flutter; L, left; R, right; TIA, transient ischemic attack.
